# A case of Henoch-Schonlein Purpura with dilated coronary arteries

**DOI:** 10.1186/s12969-018-0270-9

**Published:** 2018-09-04

**Authors:** Jessica L. Bloom, Jeffrey R. Darst, Lori Prok, Jennifer B. Soep

**Affiliations:** Department of Pediatric Rheumatology, Children’s Hospital Colorado, 13123 East 16th Avenue, B311, Aurora, CO 80045 USA

**Keywords:** Henoch-Schonlein Purpura (HSP), Dilated coronary arteries, Coronary artery dilation, Vasculitis, Arthritis, Kawasaki disease (KD), IgA, Purpura, Petechiae, Abdominal pain

## Abstract

**Background:**

Henoch-Schonlein Purpura (HSP) is one of the most common vasculitides of childhood, with 10–20 cases per 100,000 children. It frequently occurs following an infectious trigger and involves IgA and C3 deposition in small vessel walls. HSP is characterized by palpable purpura plus IgA deposition on biopsy, arthritis/arthralgia, renal involvement (hematuria and/or proteinuria), and/or abdominal pain. It is not generally recognized as a cause of dilated coronary arteries.

**Case presentation:**

We describe the first reported case of HSP presenting with dilated coronary arteries. This patient is a nine-year-old previously healthy Caucasian male who presented with 1 week of petechiae on his lower legs, knee and ankle arthritis, and abdominal pain without fever, consistent with HSP. An echocardiogram revealed coronary dilation, including the left main (5.32 mm, Z score + 4.25) and left anterior descending (LAD) (3.51 mm, Z score + 2.64) coronary arteries. He received high dose aspirin, IVIG, and infliximab with normalization of the LAD. Skin biopsy revealed leukocytoclastic vasculitis with positive IgA staining. He was Rhinovirus/Enterovirus positive with Group A Streptococcus on throat culture.

**Conclusion:**

Cardiac findings, while rare, can exist in HSP. Coronary dilation appeared to respond to our hospital protocol’s Kawasaki Disease (KD) therapy, possibly indicating an overlap in HSP and KD pathophysiology. This case, along with prior reports of dilated coronaries in systemic juvenile idiopathic arthritis (SJIA), highlights the importance of considering other sources of systemic inflammation, in addition to KD, when coronary dilation is identified. The appropriate therapy, follow-up, and prognosis for our patient are not clear, as further studies are needed to determine the natural course of these findings.

## Background

Henoch-Schonlein Purpura (HSP) is one of the most common vasculitides of childhood, with an incidence of 10–20 cases per 100,000 children. It occurs most often between the ages of three and 15 years old. HSP is more common in males than females and occurs most often between autumn and spring, frequently following an infectious trigger such as beta-hemolytic streptococcus. The pathogenesis is thought to involve a dysregulated immune response that results in an inflammatory state with IgA and C3 deposition in small vessel walls [1–3].

Clinical criteria for diagnosis, as defined in 2006 by the European League Against Rheumatism and the Pediatric Rheumatology European Society, requires palpable purpura along with one of the following: predominant IgA deposition on biopsy, arthritis or arthralgia, renal involvement (hematuria/proteinuria), or abdominal pain [4]. The diagnosis can be made on history and physical exam alone, as no specific laboratory test or imaging is diagnostic [5].

Apart from management of glomerulonephritis when present, treatment is focused on supportive care with occasional use of steroids for severe intestinal involvement or persistent arthritis. Mortality is less than 1% with long-term outcomes dependent on the extent of kidney involvement [1, 6, 7].

Few reports of HSP with cardiac involvement are described in children and none with coronary artery dilation [8–30]. Rather, Kawasaki Disease (KD) is the most common cause of acquired cardiac disease in children in the developed world. Diagnosis of KD is characterized by 5 days of fever, and four criteria among: bilateral conjunctival injection, oropharyngeal mucous membrane changes, peripheral extremity changes, polymorphous rash, and cervical lymphadenopathy > 1.5 cm [4]. Up to 25% of untreated patients develop coronary artery aneurysms, although the mechanism is unknown. Incomplete, or “atypical” KD, which presents with 5 days of fever but less than four of the above criteria, can also result in coronary involvement and is often an elusive diagnosis [31]. Surveillance of coronary arteries is standard of care, with serial echocardiograms performed during the acute and convalescent phases. Treatment of coronary involvement in the acute phase often includes high-dose aspirin, intravenous immunoglobulin (IVIG), and/or infliximab [1]. Coronary dilation can also be seen in systemic juvenile idiopathic arthritis (SJIA); however, unlike KD, the dilation is not usually treated and tends to self-resolve [32].

While reported in KD and SJIA, there have been no reports of dilated coronaries in HSP [32, 33]. Here, we present the first reported case of HSP with dilated coronary arteries.

## Case presentation

A nine-year-old Caucasian male with no significant past medical history, family history, medications, or allergies presented for an adenoidectomy due to hypertrophy. Two days later (day one of illness), he had a low-grade fever for 2 days, followed by 2 days of headache. Transient abdominal pain was noted on day five. On day six, he had swelling, warmth, tenderness, and decreased range of motion of his left knee and left foot. He was unable to walk and presented to the Emergency Department. Scattered petechiae were noted on his lower legs bilaterally. Left knee and foot x-rays were negative for fracture. CRP was 1.2 mg/dL (nl < 1) with a normal serum creatinine, complete blood count, and urinalysis. He was discharged home with supportive care for presumed Henoch-Schonlein Purpura.

On day nine, he saw his primary care physician (PCP) for follow-up. He continued to have intermittent swelling of his bilateral knees and ankles, low-grade temperatures (99-100F), and a non-blanching rash on his lower extremities. A slightly red posterior oropharynx was noted, however rapid throat swab for Group A streptococcus was negative. Later that day, he developed severe abdominal pain and returned to the Emergency Department.

Upon arrival, he was afebrile and received fentanyl for pain control. Laboratory results included CRP 5.1 mg/dL (0–1), ESR 10 mm/hr. (0–15), ASO antibody 530 IU/mL (0–200), Anti-DNase B antibody 588 U/mL (0–170), negative ANA, RF 6.5 IU/mL (0–13.9), C4 44 mg/dL (14–44), C3 183 mg/dL (82–167), and positive Rhinovirus/Enterovirus from a nasal washing. Gamma-glutamyl transferase level, complete metabolic panel, complete blood count, blood cultures, and urinalysis were all unremarkable.

**Fig. 1:**
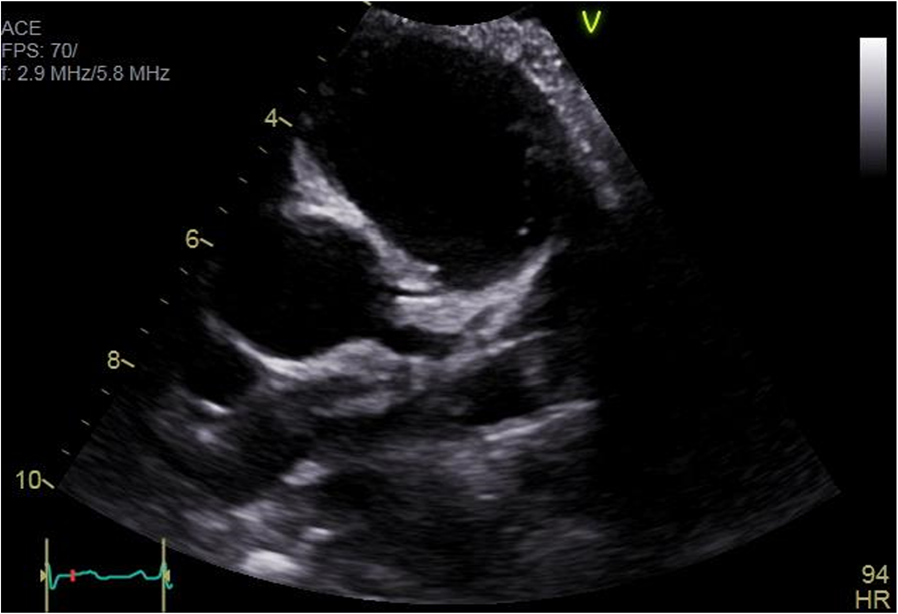
Echocardiogram. Initial echocardiogram demonstrating dilation of the left main coronary artery (5.32 mm, Z score + 4.25)

**Fig. 2:**
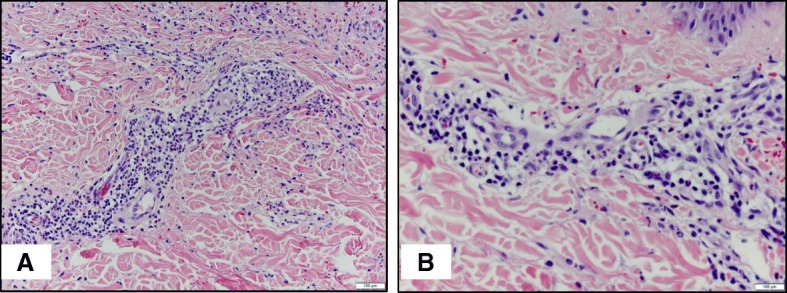
Skin Biopsy. **a** 20× view of vessels with thickened and homogenized lumens and surrounding acute inflammation (neutrophil predominant, with rare eosinophils). All are hallmarks of leukocytoclastic vasculitis seen in HSP. Note surrounding dermal hemorrhage. **b** 40X view. Granular staining of IgA in vessels was noted under immunofluorescence

Abdominal ultrasound suggested mild bowel wall thickening in the left upper quadrant at the site of point tenderness. An echocardiogram, ordered by his PCP to rule out acute rheumatic fever, revealed dilation of the left main coronary artery (LMCA: 5.32 mm, Z score + 4.25) and left anterior descending coronary artery (LAD: 3.51 mm, Z score + 2.64) with no other abnormalities (Fig. 1). EKG was unremarkable. He ultimately had a skin biopsy of his lower extremity rash that revealed leukocytoclastic vasculitis with positive IgA staining (Fig. 2).

The patient met criteria for HSP given his palpable purpura, abdominal pain, arthritis and skin biopsy. He did not meet criteria for typical (or atypical) KD, given his lack of persistent fever. Nevertheless, KD treatment was initiated, per Infectious Disease and Cardiology, given the potential sequelae of untreated inflammatory-mediated dilation of the coronaries. He received high dose aspirin, 2 g/kg of IVIG over 10 h, and 5 mg/kg of infliximab as per our hospital protocol. His CRP normalized and his abdominal pain improved. He was discharged with Cardiology follow-up for an echocardiogram and a PCP appointment to monitor blood pressure and kidney function. Of note, Group A streptococcus culture turned positive during his admission and thus amoxicillin was started.

By day 17, his arthritis resolved, his rash improved, and his energy and appetite returned. Repeat echocardiogram on day 21 showed persistent dilation of his LMCA (5.24 mm, Z score + 3.91) and LAD (3.74 mm, Z Score + 3.07). He remained on daily low dose aspirin until his next echocardiogram 3 months later revealed normal coronary dimensions. He had no evidence of hypertension, hematuria or proteinuria.

## Discussion and conclusions

This is the first reported case of HSP with dilated coronary arteries. This patient met criteria for HSP and likely had dilation of his arteries due to the vasculitis. We considered multiple other diagnoses, as described below, as there were no published reports of coronary artery dilation in HSP; however, his clinical picture did not fit these other entities.

We considered the possibility of simultaneous KD and HSP. However, he did not meet criteria for typical or atypical KD. There have only been three published cases of patients diagnosed with concurrent HSP and KD. One patient, described by Miura et al., had KD, followed by HSP and then two subsequent occurrences of simultaneous KD and HSP [34]. Vedagiriswaran et al. described a five-year-old who presented with concurrent HSP and KD, including dilated coronary arteries [35]. Heldrich et al. discussed a three-year-old who presented with KD, developed Hemolytic Uremic Syndrome on day three, and then HSP by week two [36].

Additionally, our patient did not have any remote history resembling KD to suggest pre-existing cardiac dilation. There have only been three reported cases of HSP in patients with prior KD, of which only one patient had dilated coronary arteries as a result of his KD [34, 37, 38]. Further, interval improvement on echocardiogram after treatment would have been unlikely had our patient’s dilated arteries been longstanding.

Acute Rheumatic Fever in addition to his HSP was considered, however he did not meet criteria. He grew group A streptococcus on throat culture, but this is a known trigger for HSP. There is one reported case of coronary artery dilation with Acute Rheumatic Fever; notably, that patient also had valvular involvement and did not have HSP [39]. SJIA can also lead to dilated coronary arteries in childhood, however our patient did not meet diagnostic criteria [32]. One might also consider Polyangiitis Overlap Syndrome as described by Fauci and Leavitt, however he only met criteria for one type of vasculitis [40, 41].

Our patient’s coronary dilation was found serendipitously, but highlights the importance of considering various causes of systemic inflammation when dilated coronary arteries are identified. While we hoped to improve his outcome with aspirin, IVIG, and infliximab, we cannot be certain of the natural course had no intervention been pursued. If the treatment did improve his coronary dilation, it may indicate an overlap of the inflammatory pathophysiology of HSP and KD as they are both forms of vasculitis. It is often most practical to consider childhood vasculitides as unique conditions with concrete findings, however this patient’s presentation suggests the possibility of overlap between different forms.

It is important to note that, while cardiac complications are rarely considered in HSP, there have been approximately 20 published cases of HSP with non-coronary artery cardiac involvement. Interestingly, not all patients had cardiac symptoms, and patients with HSP-related cardiac involvement are generally adults. Myocardial biopsies in some have shown small vessel leukocytoclastic vasculitis and necrosis [8–13]. One biopsy revealed granular deposits of IgA and C3 within intramyocardial vessel walls, which resolved after 5 months of cyclophosphamide and prednisone [8]. There have been a few cases of arrhythmias with HSP, including complete heart block and a slow junctional rhythm, one of which resolved with steroid treatment [12, 14–16]. Six cases of myocardial ischemia and/or infarctions during or following HSP have been reported in patients who lacked other cardiovascular risk factors, thought to be secondary to HSP-related vasculitis [17–21]. Michas described a 19-year-old with acute heart failure from myocarditis in the setting of HSP [22]. Five HSP cases had rheumatic carditis thought to be due to co-existing Acute Rheumatic Fever [23–28]. Two patients with severe mitral regurgitation and pulmonary hemorrhage in the setting of HSP have been described, but the etiology is not well understood [29, 30]. This may be a distinction in how HSP presents in adults, but could also be influenced by an increased likelihood to undergo cardiac evaluation.

Given that a cardiac work-up is not standard of care for HSP, our case raises the question of whether coronary artery dilation is more prevalent than previously considered. As it stands, performing an EKG and echocardiogram on all patients with HSP is not recommended. Further studies to determine the prevalence of coronary artery dilation in children with HSP and, if present, the natural course of the coronary changes could aid in this decision-making.

## Abbreviations

HSP: Henoch-Schonlein Purpura
IVIG: Intravenous immunoglobulin
KD: Kawasaki Disease
LAD: Left anterior descending
LMCA: Left main coronary artery
PCP: Primary care provider
SJIA: Systemic juvenile idiopathic arthritis
